# Construction of the First SNP-Based Linkage Map Using Genotyping-by-Sequencing and Mapping of the Male-Sterility Gene in Leaf Chicory

**DOI:** 10.3389/fpls.2019.00276

**Published:** 2019-03-11

**Authors:** Fabio Palumbo, Peng Qi, Vitor Batista Pinto, Katrien M. Devos, Gianni Barcaccia

**Affiliations:** ^1^Laboratory of Genomics for Plant Breeding, Department of Agronomy Food Natural Resources Animals and Environment (DAFNAE), University of Padova, Legnaro, Italy; ^2^Institute of Plant Breeding, Genetics and Genomics, Department of Plant Biology, University of Georgia, Athens, GA, United States; ^3^Institute of Plant Breeding, Genetics and Genomics, Department of Crop and Soil Sciences, University of Georgia, Athens, GA, United States; ^4^Department of General Biology, Federal University of Viçosa, Viçosa, Brazil

**Keywords:** *Cichorium intybus*, genetic linkage map, male sterility, *ms1* locus, single nucleotide polymorphism (SNP) markers, genotyping-by-sequencing (GBS), transcription factor MYB103

## Abstract

We report the first high-density linkage map construction through genotyping-by-sequencing (GBS) in leaf chicory (*Cichorium intybus* subsp. *intybus* var. *foliosum*, 2*n* = 2*x* = 18) and the SNP-based fine mapping of the linkage group region carrying a recessive gene responsible for male-sterility (*ms1*). An experimental BC_1_ population, segregating for the male sterility trait, was specifically generated and 198 progeny plants were preliminary screened through a multiplexed SSR genotyping analysis for the identification of microsatellite markers linked to the *ms1* locus. Two backbone SSR markers belonging to linkage group 4 of the available *Cichorium* consensus map were found genetically associated to the *ms1* gene at 5.8 and 12.1 cM apart. A GBS strategy was then used to produce a high-density SNP-based linkage map, containing 727 genomic loci organized into 9 linkage groups and spanning a total length of 1,413 cM. 13 SNPs proved to be tightly linked to the *ms1* locus based on a subset of 44 progeny plants analyzed. The map position of these markers was further validated by sequence-specific PCR experiments using an additional set of 64 progeny plants, enabling to verify that four of them fully co-segregated with male-sterility. A mesosynteny analysis revealed that 10 genomic DNA sequences encompassing the 13 selected SNPs of chicory mapped in a peripheral region of chromosome 5 of lettuce (*Lactuca sativa* L.) spanning about 18 Mbp. Since a MYB103-like gene, encoding for a transcription factor involved in callose dissolution of tetrads and exine development of microspores, was found located in the same chromosomal region, this orthologous was chosen as candidate for male-sterility. The amplification and sequencing of its CDS using accessions with contrasting phenotypes/genotypes (i.e., 4 male sterile mutants, *ms1ms1*, and 4 male fertile inbreds, *Ms1Ms1*) enabled to detect an INDEL of 4 nucleotides in its second exon, responsible for an anticipated stop codon in the male sterile mutants. This polymorphism was subsequently validated through allele-specific PCR assays and found to fully co-segregate with male-sterility, using 64 progeny plants of the same mapping BC_1_ population. Overall, our molecular data could be practically exploited for genotyping plant materials and for marker-assisted breeding schemes in leaf chicory.

## Introduction

Linkage maps based on molecular markers play a key role in the study of the genetics and genomics of crop plants. Among the possible applications, the development of high-density linkage maps has simplified the discovery of Mendelian genes (Kaur et al., 2014; Zhao et al., 2015; Huang et al., 2016) and quantitative trait loci (QTL) (Colasuonno et al., 2014; Marcotuli et al., 2017; Schumann et al., 2017). The first genetic linkage map of chicory (*Cichorium intybus* subsp. *intybus* L.), a leafy vegetable crop belonging to the family Asteraceae and widely cultivated in many European countries, consisted of 431 SSR and 41 EST markers, and covered 878 cM (Cadalen et al., 2010).

Chicory is a diploid plant species (2*n* = 18) that is naturally allogamous, due to an efficient sporophytic self-incompatibility system (Barcaccia et al., 2003; Lucchin et al., 2008). In addition, outcrossing is promoted by a number of traits, including: (i) a floral morpho-phenology (i.e., proterandry, with the anthers maturing before the pistils) unfavorable to selfing in the absence of pollen donors (Pécaut, 1962; Desprez et al., 1994); and (ii) a competitive advantage of allo-pollen grains and tubes (i.e., pollen genetically diverse from that produced by the seed parents) (Desprez and Bannerot, 1980; Eenink, 1982). Two main botanical varieties can be recognized within *C. intybus* subsp. *intybus* to which all the cultivated types of chicory belong. The first is var. *foliosum*, which traditionally includes Witloof chicory, Pain de sucre, Catalogne and Radicchio and all the cultivar groups whose commercial products are the leaves (i.e., leaf chicory). The second is var. *sativum* and comprises all the types whose commercial product, either destined to industrial transformation or direct human consumption, is the root (i.e., root chicory). In root chicory, Gonthier et al. (2013) identified molecular markers associated with the Nuclear Male-Sterility 1 (NMS1) locus and the Sporophytic Self-Incompatibility (SSI) locus. These two loci were both mapped to narrow genomic regions belonging, respectively, to linkage groups 5 and 2 of the genetic map developed by Cadalen et al. (2010). Similarly, in leaf chicory, Barcaccia and Tiozzo Caenazzo (2012, 2014) mapped molecular markers linked to the male-sterility gene (*ms1*) within linkage group 4, according to the map by Cadalen et al. (2010).

Recently, a chicory genetic linkage map spanning 1,208 cM was developed by Muys et al. (2014) using an F_2_ population composed of 247 plants. This map comprised 237 markers (i.e., 170 AFLP, 28 SSR, 27 EST-SNP, and 12 EST-SSR markers) and covered about 84% of the chicory genome. The markers were then used to find potential orthologous based on sequence homology in mapped lettuce EST clones from the Compositae Genome Project Database (Muys et al., 2014). A total of 27 putative orthologous pairs were retained, pinpointing seven potential blocks of synteny that covered 11% of the chicory genome and 13% of the lettuce genome, opening new avenues for the comparative analysis of these two species.

Mapping of the self-incompatibility and male-sterility mechanisms in chicory is important, not only to understand the genetic basis of the main reproductive barriers that act in flowering plants, but also because of the potential applications of these loci for breeding F_1_ hybrid varieties. In fact, although in the past chicory varieties were mainly synthetics produced by intercrossing a number of phenotypically superior plants, selected on the basis of morpho-phenological and commercial traits, recently private breeders and seed firms have developed methods for the development of F_1_ hybrids.

In the last century, male sterile mutants have allowed the exploitation of heterosis (i.e., hybrid vigor) through the development of F_1_ hybrid varieties in many agricultural and horticultural crops. In general, male-sterility is defined as the failure of plants to develop anthers or to form functional pollen grains and it is more prevalent than female-sterility. In nature, male sterile plants have reproduction potentials because they can still set seeds, as female-fertility is unaffected by most of the mutations responsible for male-sterility. This behavior is known to occur spontaneously via mutations in nuclear and/or cytoplasmic genes involved in the development of anthers and pollen grains. Barcaccia and Tiozzo Caenazzo (2012, 2014) have recently identified and characterized a spontaneous male sterile mutation in cultivated populations of leaf chicory, namely Radicchio (*C. intybus* subsp. *intybus* var. *foliosum* L.). Cytological analyses revealed that microsporogenesis proceeds regularly up to the development of tetrads when the microspores arrest their developmental program showing a collapse of the exine. At the beginning of microgametogenesis, non-viable shrunken microspores were clearly visible within anthers. Moreover, genetic segregation data derived from replicated F_2_ and BC_1_ populations clearly supported a nuclear origin, monogenic control and recessive nature of the male-sterility trait in the leaf chicory mutants (Barcaccia and Tiozzo Caenazzo, 2012, 2014).

In this work, taking advantage of the method of genotyping based on 27 mapped microsatellite marker loci scattered throughout the linkage groups of leaf chicory (Ghedina et al., 2015) and the first genome sequence draft of leaf chicory with the functional annotation of more than 18,000 unigenes (Galla et al., 2016), we successfully constructed a high-density linkage map and finely mapped the *ms1* locus in leaf chicory. After a preliminary genetic mapping of the *ms1* locus using SSR and EST markers, a Genotyping-By-Sequencing (GBS) methodology was used to narrow down the chromosomal window around the *ms1* gene, first of all developing well-saturated linkage groups for this species and then selecting molecular markers and candidate genes for male-sterility exploitable for marker-assisted breeding and gene cloning programs.

## Materials and Methods

### Plant Materials and Genomic DNA Extraction

**FIGURE 1:**
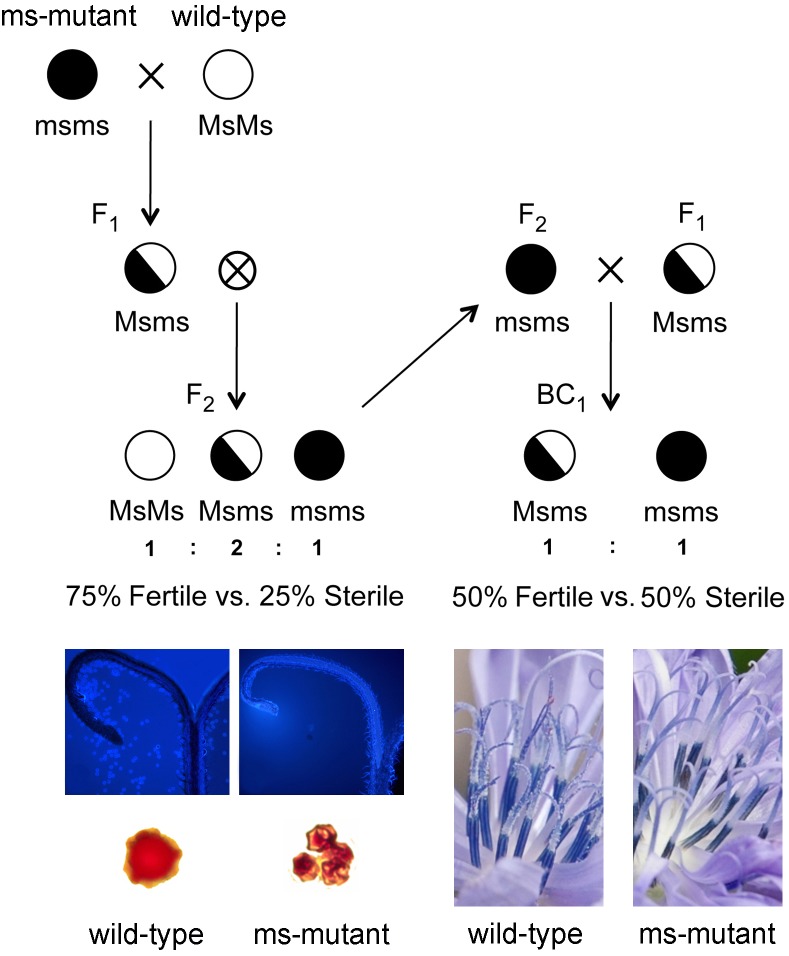
Schematic representation of the breeding populations developed for mapping the gene responsible for male-sterility in *Cichorium intybus*. A male-sterile mutant plant (genotype *msms*), belonging to a cultivated population of radicchio was crossed with a male-fertile plant (genotype *MsMs*) selected from a spontaneous accession of wild chicory. An F_1_ male-fertile plant heterozygous at the male-sterility locus (genotype *Msms*) was selfed and an F_2_ male-sterile plant (genotype *msms*) of the segregating population was then backcrossed as seed parent to a sister F_1_ male-fertile plant (genotype *Msms*) used as pollen donor. Individuals of the BC_1_ population were phenotyped by visual observations of anthers (*in vivo* screening for the presence/absence of pollen) and cytological investigations (*in vitro* staining of pollen using acetocarmine and DAPI solutions; for detailed information on pollen viability analysis please see Barcaccia and Tiozzo Caenazzo, 2012). At flowering, anthers were preliminary screened for the absence vs. presence of pollen and microscopy was then used to validate the sterile vs. fertile phenotype (mutants were characterized by shapeless, smaller and shrunken microspores as compared to the wild-type ones).

Several male sterile mutants sharing the same genotype at the *ms1* locus were discovered by T&T Srl Agricola (Blumen Group SpA) within local varieties of radicchio “Red of Chioggia” stemmed from recurrent phenotypic selection programs (Barcaccia and Tiozzo Caenazzo, 2012, 2014). A backcross (BC_1_) population segregating 1:1 for the male-sterility trait and comprising 198 individual plants was generated as follows. A male-sterile mutant plant (genotype *msms*), belonging to a cultivated population of radicchio was crossed with a male-fertile plant (genotype *MsMs*) selected from a local accession of wild chicory, in order to maximize genetic diversity and polymorphism levels. An individual F_1_ male-fertile plant heterozygous at the male-sterility locus (genotype *Msms*) was selfed and one F_2_ male-sterile progeny (genotype *msms*) was then backcrossed as seed parent to a sister F_1_ male-fertile plant (genotype *Msms*) used as pollen donor (Figure 1). The agronomic field trials for plant phenotyping (discrimination of male-fertile and male-sterile phenotypes) were conducted at the experimental farm “Lucio Toniolo” of the University of Padova, located in Legnaro (Padova, Italy) (45°21′5,68″N 11°57′2,71″E – 8 m above the sea level). Seeds were sown in February and grown in heated greenhouse, under light/dark cycle conditions of 12/12 h and temperature of 20 °C. Uniformly sized, 4 week-old seedlings were transplanted in the field under polyethylene tunnels and under controlled pollination conditions. The soil texture was the following: 46 sand, 24% clay and 30% loam; pH = 7.9; electric conductibility 112 μS; organic carbon 1.1%. Flowering started around 150 days after sowing and all individuals of the BC_1_ population were phenotyped using three flowers per plant by visual observations of the anthers (*in vivo* screening for the presence/absence of pollen) and cytological investigations (*in vitro* staining of pollen). More in details, cytological investigations were accomplished using both aceto-carmine and DAPI solutions. The aceto-carmine staining technique was used to measure discriminant phenotypic descriptors, such as shape, size and coloration of pollen grains, as reported by Janssen and Hermsen (1976) and implemented by Barcaccia et al. (1998), while DAPI staining was accomplished as described by Barcaccia and Tiozzo Caenazzo (2012). Microscopy characterized a mutant phenotype by shapeless, small and shrunken microspores as compared to the wild-type ones (Figure 1). It is worth mentioning that in mutant plants at the stage of dehiscent anthers, microspores were arrested in their development at the uninucleate stage, and collapsed before their release from the tetrads. Viable pollen grains were never detected in mature anthers, demonstrating full expression of the male-sterility trait (Barcaccia and Tiozzo Caenazzo, 2012, 2014).

Total genomic DNA of the parents and progeny was isolated from 100 mg of fresh leaf tissue using the DNeasy^®^ Plant mini-kit (Qiagen, Hilden, Germany) following the recommendations of the manufacturer. Quality and concentration of DNA samples were estimated by spectrophotometric analysis (NanoDrop 2000c UV-Vis, Thermo Fisher Scientific, San Jose, CA, United States) and quality was also assayed by agarose gel electrophoresis (1.0% w/v agarose TAE 1× gel containing 1× SYBR^®^ Safe, Thermo Fisher Scientific).

### Preliminary Genetic Mapping of the *ms1* Locus With Simple Sequence Repeat (SSR) and Cleaved Amplified Polymorphic Sequence (CAPS) Markers

The entire BC_1_ population of leaf chicory was used for genetic mapping of the male-sterility gene using three selected SSR markers (M4.12, M4.11b, and M4.10b) and one EST-derived CAPS marker previously mapped on linkage group 4 (Cadalen et al., 2010; Barcaccia and Tiozzo Caenazzo, 2012, 2014). This step was preliminarily applied in order to determine and validate upstream and downstream backbone DNA markers encompassing the *ms1* locus.

An AFLP-derived amplicon corresponding to marker E02M09 (Barcaccia and Tiozzo Caenazzo, 2012), whose sequence was found to encompass a microsatellite region (GenBank accession JF748831), was converted into a SSR marker and renamed as M4.12 by Ghedina et al. (2015). This sequence-tagged site marker was mapped on linkage group 4 and was therefore included in the genetic analysis of the BC_1_ population. Among the microsatellite marker loci publicly available for the leaf chicory genome (Cadalen et al., 2010) and associated to the linkage group 4, the M4.11b (Ghedina et al., 2015) (synonym EU03H01) contained an imperfect [(TG)_5_CG(TG)_7_] microsatellite motif (GenBank accession KF880802) and M4.10b (Ghedina et al., 2015) [synonym EU07G10 (Cadalen et al., 2010)] carried a (CT)_8_TT(CT)_5_CC(CT)_3_TT(CT)_7_ microsatellite motif (GenBank accession KX534081).

For SSR amplification, the three-primer strategy reported by Schuelke (2000) was adopted, with some modifications. Briefly, forward primers were tagged at their 5′ end with universal sequences (M13 or PAN2, unpublished) and used in PCR reactions in combination with sequence-specific reverse primers, and M13 and PAN2 oligonucleotides labeled with the fluorophores 6-FAM and NED, respectively.

The PCR reactions were performed in a total volume of 10 μl containing approximately 20 ng of gDNA template, 1× Platinum^®^ Multiplex PCR Master Mix (Applied Biosystems, Carlsbad, CA, United States), GC enhancer 10% (Applied Biosystems), 0.05 μM tailed forward primer (Invitrogen Corporation, Carlsbad, CA, United States), 0.1 μM reverse primer (Invitrogen Corporation) and 0.23 μM universal primer (Invitrogen Corporation). The following thermal conditions were adopted for all reactions: 2 min at 95°C for the initial denaturation step, 45 cycles at 95°C for 30 s, 55°C for 30 s and 72°C for 45 s. A final extension step at 72°C for 30 min terminated the reaction, to fill-in any protruding ends of the newly synthesized strands.

Amplicons were initially separated and visualized on 2% agarose gels in 1× TAE gel containing 1× Sybr Safe DNA stain (Life Technologies, Carlsbad, CA, United States). The remainder of the fluorescent labeled PCR products (8 μl) was subjected to capillary electrophoresis on an ABI PRISM 3130xl Genetic Analyzer (Thermo Fisher). LIZ500 (Applied Biosystems) was used as molecular weight standard.

The CAPS marker was developed from a MADS-box gene (GenBank accession AF101420) which was initially considered (Cadalen et al., 2010) but later disproven (Barcaccia et al., 2016) to be a candidate gene for male-sterility. Several primer pairs were designed for nested PCR assays using PerlPrimer v1.1.21 and used to amplify the full-length sequence and sub-regions of the MADS-box gene from plants of the BC_1_ population phenotyped for male-sterility. Amplification reactions were performed in a 9700 Thermal Cycler (Applied Biosystems) with the following conditions: initial denaturation at 94°C for 5 min followed by 30 cycles at 94°C for 30 s, 57°C for 30 s, 72°C for 60 s and a final extension of 10 min at 72°C, and then held at 4°C. The quality of PCR products was assessed on a 2% (w/v) agarose gel stained with 1× SYBR^®^ Safe^TM^ DNA Gel Stain (Life Technologies). Amplicons were Sanger-sequenced to detect SNPs potentially associated with male-sterility and to locate restriction sites. PCR products were then cleaved using *Nco*I (Promega, Madison, WI, United States) as endonuclease specific for single nucleotide variants, following the protocol suggested by the manufacturer. Amplicons were digested at 37°C for 2 h. CAPS variants were visualized on 2.5% (w/v) agarose gels (Life Technologies) stained with 1× SYBR^®^ Safe^TM^ DNA Gel Stain (Life Technologies).

Segregation data from the three SSR markers and the MADS-box gene-specific CAPS marker were analyzed with JoinMap^®^ v. 2.0 (Stam, 1993) using the BC_1_ population type option. Genetic association between each of the markers and the male-sterility trait was assessed by recording the target *ms1* locus as a qualitative trait. The grouping module was applied with a LOD threshold of 3 and a maximum recombination frequency *r* of 40%. The genetic distance between each pair-wise comparison of marker locus and target locus, expressed in centiMorgans (cM), was calculated from the recombination frequency corrected with the Kosambi’s mapping function (Kosambi, 1943). MapChart v.2.3 (Voorrips, 2002) was used to display the map.

### Construction of a SNP-Based Linkage Map Using Genotyping-by-Sequencing (GBS)

Genomic DNA from 22 male sterile progeny and 22 male fertile progeny from the BC_1_ population, along with 2 DNA samples from the parental plants were quantified with the dsDNA BR assay on a Qubit^®^ 1.0 fluorometer (Invitrogen, Carlsbad, CA, United States) and DNA concentrations were normalized to 20 ng/μl. DNA samples were shipped to LGC Genomics (Berlin, Germany) for GBS library preparation, sequencing and subsequent bioinformatic analysis. Briefly, DNA samples were digested with the restriction enzyme *Msl*I (NEB, Beverly, MA, United States) and the indexed Illumina libraries were prepared using the Ovation Rapid DR Multiplex System (Nugen, Leek, Netherlands) according to the protocol provided by the manufacturer. The 46 libraries were pooled removing PCR primers and small amplicons by Agencourt XP bead purification (Beckman Coulter, High Wycombe, United Kingdom) and then normalized using the Trimmer Kit (Evrogen, Moscow, Russia). The normalized library pool was amplified using MyTaq polymerase (Bioline, Taunton, MA, United States) and standard Illumina TrueSeq amplification primers (Illumina Inc., San Diego, CA, United States). The nGBS library was finally size selected on a LMP-Agarose gel, removing fragments smaller than 300 bp and larger than 500 bp, and sequenced on a single lane of an Illumina NextSeq 500 v2 (2 × 150 bp, Illumina Inc., San Diego, CA, United States).

Raw reads were de-multiplexed and split according to their barcodes using Illumina bcl2fastq 2.17.1.14 software (Illumina, San Diego, CA, United States) and sequencing adapter remnants were clipped. After this step, using proprietary LGC Genomics software reads were processed as follows: (i) trimming of the 3′-end (to get a minimum average Phred quality score of 20 over a window of ten bases); (ii) discarding reads with 5′-ends not matching the restriction enzyme site; (iii) removing all reads containing undetermined (N) bases; (iv) discarding reads with final length < 64 bases. CD-HIT EST (Fu et al., 2012) clustered all the processed sequences in a reference catalog of consensus loci, allowing up to 5% difference. BWA v.0.7.12^1^ was used to align the reads from each sample against the newly constituted reference catalog and Freebayes v1.0.2-16^2^ was then employed at default settings for variant discovery. The raw SNP variants were filtered by applying customized Perl scripts according to the following rules: (i) minimum allele count exceeding eight reads; (ii) allele frequency across all samples between 5 and 95%; (iii) genotypes observed in at least 32 samples; (iv) discard adjacent SNPs and SNPs with more than two alleles (i.e., only biallelic SNPs were taken into account).

Segregation data, analyzed using a modified version of MapMaker v3 software^3^ consisted of GBS-SNP data, genotypic scores for SSR markers M4.10b, M4.12, M2.6, and M2.4 (Ghedina et al., 2015) and qualitative scores for the male-sterility locus *ms1*. SSRs M2.6 and M2.4 were included because they have been shown by Gonthier et al. (2013) to be associated with the self-incompatibility locus. Linkage groups were formed at a LOD threshold of 5. Marker orders within single linkage groups were determined using the MapMaker functions ‘order,’ ‘try,’ and ‘ripple,’ and were checked manually to ensure optimal placement of the marker loci. Genetic distances were calculated using the Kosambi mapping function and graphically represented using MapChart v2.3 (Voorrips, 2002).

The newly developed genetic map was enriched by locating markers on the first genome draft of leaf chicory (Galla et al., 2016) using a BLASTN approach (similarity >90%, *E*-value < 1E-50). Putative functional annotation of genes present in the mapped contigs was performed using BLASTX against the TAIR10 (Berardini et al., 2015) database (*E*-value < 1E-5).

### Validation of SNP Variants Linked to the Male-Sterility Locus Through Allele Specific (AS)-PCR Assays

All SNPs potentially associated with the male-sterility *ms1* locus and exhibiting a maximum of three recombinant events with *ms1* were validated in a larger number of progeny through allele-specific PCR (AS-PCR) assays.

A total of 64 genomic DNA samples (i.e., 32 male sterile plants and 32 male sterile plants) from the same BC_1_ population but not included in the GBS analysis were used for amplification using two sets of primers for each male-sterility associated SNP. Each primer set consisted of a different allele-specific forward primer and a common locus-specific reverse primer. The two allele-specific PCR primers were designed so that the 3′ nucleotide was complementary to one allele of the putative polymorphism. Amplicons were produced were in a 9700 Thermal Cycler (Applied Biosystems) with the following conditions: initial denaturation at 94°C for 5 min followed by 30 cycles at 94°C for 30 s, 55°C for 30 s, 72°C for 60 s and a final extension of 10 min at 72°C, and then held at 4°C. PCR products were separated on 2.0% w/v agarose TAE 1× gels containing 1× SYBR^®^ Safe stain (Thermo Fisher Scientific). Segregation data for *ms1* and *ms1*-associated markers obtained in the entire set of 108 (44 used in GBS + 64 used in AS-PCR) BC_1_ progeny were used to build a new genetic linkage map for the chromosomal block carrying the target locus with JoinMap^®^ v. 2.0 (Stam, 1993). The BC_1_ population type option was adopted and the Kosambi mapping function was used to calculate genetic distances. The resulting map was drawn with MapChart v.2.3 (Voorrips, 2002).

### Micro-Synteny Comparison Between *Lactuca sativa* and *Cichorium intybus* Homologous Chromosomal Segments

Considering that the recent release of the high quality *Lactuca sativa* genome assembly (Reyes-Chin-Wo et al., 2017) represents a reference assembly for the whole Asteraceae family, a BLASTN approach (*E*-value < 1E-20) was performed aligning the chicory contigs carrying the 13 SNPs associated to the *ms1* locus against the genome of lettuce. Robust evidences of micro-synteny between these two species, made MYB103 worth to be investigated due to its possible functional involvement in the male sterility (Zhang et al., 2007). Since contig_119275 from the chicory genome draft, showed the best match with Lsat_1_v5_gn_5_561 locus, annotated in *L. sativa* as MYB103, it was used to design three couple of primers, covering the entire coding DNA sequence (CDS, Table 1). Amplicons of four commercial male sterile accessions (namely D49, A89, B86, and D17) and four male fertile inbreed lines (namely 2111, 202, 231, and 334) were Sanger-sequenced to detect polymorphisms between these two groups with contrasting microgametogenesis. According to a four nucleotides insertion observed in all male sterile accessions within the putative MYB103 gene, two additional couples of primer were used in an AS-PCR assay to test the 64 genomic DNA samples (i.e., 32 male sterile plants and 32 male sterile plants) from the BC_1_ population previously used to validate the *ms1*-associated SNPs (Table 1). In details two allele-specific forward primers were designed so that one amplified the wild type allele (without the insertion) while the other amplified only the mutated allele (with the insertion). Two reverse primers were designed within a conserved region of the second exon in order to produce amplicons of different size, distinguishable by standard agarose gel electrophoresis (i.e., MYB103_MS and MYB103_MF, Table 1). The two PCR reactions specifically designed for the wild type and the mutated alleles were performed separately for each DNA sample at the same conditions used for the previous AS-PCR analysis. The two PCR outputs of each individual were mixed and run together on a 2.0% w/v agarose TAE 1× gels containing 1× SYBR^®^ Safe stain (Thermo Fisher Scientific).

**Table 1:** List of primer pairs used to amplify the mapped markers and the candidate gene MYB103 of linkage group 9 [corresponding to linkage group 4 of Cadalen et al. (2010)].

Locus name	GenBank ID	Polymorphism	Forward primer	Reverse primer
EU02M09^3^ M4.12^2^	JF748831^3^	(TC)_n_	GGCATCGGGATAGAAAAACA	TCAATGCCTCAACAGAAATCC
EU03H01^1,3^ M4.11b^2^	KF880802^1,2,3^	(TG)_n_CG(TG)_n_	GCCATTCCTTTCAAGAGCAG	AACCCAAAACCGCAACAATA
EU07G10^1^ M4.10b^2^	KX534081^4^	(CT)_n_CATA/(CA)_n_CT(CA)_n_	CATCCATTATTGGGCAG	CACCAACGAACTCCTTACAAA
MADS box L2/R2^1^ CAPS marker^4^	AF101420^1^ KX45584/0/1^4^	*Nco*I	TTTTTGTGGGGTTTTGATTTTAGA	TGAGATTGCATGAATGAGAACA
T11292^4^	KX789069^4^	g.205A > C	GAATGAAAATTGACATAATC	TTTGTTGATTCTGTTCCTG
			GATGCATTAACATGGGTCT	GACTGATGGATGTCCAAT
T4403^4^	KX789070^4^	g.253A > T	GCGAACRAATGAGGATATATGAG	GTGTTGATTGAGTGTAAATCT
				GTGTTGATTGAGTGTAAATCA
T4390^4^	KX789071^4^	g.102C > T^∗^; g.139T > C	TGTAACGCCCGTAAACCCAA	GACATGTTTACTAAGGTGATGATAATATAA
				GACATGTTTACTAAGGTGATGATAATATAG
T4391^4^	KX789072^4^	g.150C > T	TAAATGTGCAATACCATGAAGC	AAGTGAGTAAGTGGTTGTATTC
			TAAATGTGCAATACCATGAAGT	
T4393^4^	KX789073^4^	g.137A > G	AACACATGAAGGMACTCTAG	GATGGGTATTGAAACTTATG
			AGGTCCCTCATATTAAAGG	GATGTTTGTGAATGATGTTT
T4392^4^	KX789074^4^	g.68C > T	TTGTTGGAAGTGATGAGGTGT	TGTTATTAACTGTGTTTCGTGATATAG
				TGTTATTAACTGTGTTTCGTGATATAA
T4399^4^	KX789075^4^	g.24T > A	AACATGATTTGTCTGCCA	CATCACAATATTCTATCCAAA
			GACATTTTTGGAACACTTTTTA	ACTGTTACATAATGGCTAG
T4394^4^	KX789076^4^	g.132T > C; g.235G > A^∗^	GTTGGTCTGTTGATTGTTGG	CATGTATATTTGGAAGTTATCAACA
				CATGTATATTTGGAAGTTATCAACG
T4401^4^	KX789077^4^	g.89T > A	GTAAAGAGTGGGAGTCATATA	CTTGTTGCCTTTGTTCTAGA
			GTAAAGAGTGGGAGTCATATT	GTTTTCAACATCMTTCACT
T4402^4^	KX789078^4^	g.166T > G	CAGTGGCAATGCACAT	ATAGTCTCATGGTATGCAGT
			GGAAGTAAATACTATACTATCGC	CATGACCACAAGGTAAACC
T4400^4^	KX789079^4^	g.231G > A	CTTAATTAGGACAGAACTTAATATAGACAG	GGAGTGGGGTTAAAGGGAA
			CTTAATTAGGACAGAACTTAATATAGACAA	
T4395^4^	KX789080^4^	g.179C > T^∗^; g.214T > C	GATTCTGCAATCGGATATCGCCTTT	ACACCGCTGATAACCACCACC
			GATTCTGCAATCGGATATCGCCTTC	
T4398^4^	KX789082^4^	g.175C > T	TGCTGCCCGGAGAAATGTTAG	TTTAGGCAACCGAAGTAAGGCAGTG
				TTTAGGCAACCGAAGTAAGGCAGTA
MYB103_exon1^4^	MK285053–MK285054	–	GAGCGAGAGTGGTGGCTAT	TTACCTCACCCAACCAGAAA
MYB103_intron1^4^		–	CAGAGTCATCGGCGTTTCTA	GTGTGAAGAAGGGTCAGTGG
MYB103_exon2^4^		–	GATCACAGGCGTCAAGGTTA	CGGAGATAGCCACCACTCT
MYB103_MS^4^	–	–	CGTTTGCTTCATGCTTAATT	AAGAACCATTGGAACACGAA
MYB103_MF^4^	–	–	CGTTTGCTTCATGCTTAGA	GGAGATAGCCACCACTCT

*^1^Cadalen et al., 2010; ^2^Ghedina et al., 2015; ^3^Barcaccia and Tiozzo Caenazzo, 2012, 2014; ^4^Present paper. The name of the locus, the GenBank accession of the trait amplified, the polymorphism observed in ms mutants and the primer pairs are reported. SNPs marked with an asterisk were not tested with allele-specific primer combinations.*

## Results

### Molecular Mapping of the SSR and CAPS Markers in the Linkage Group Carrying the Male-Sterility Locus

**FIGURE 2:**
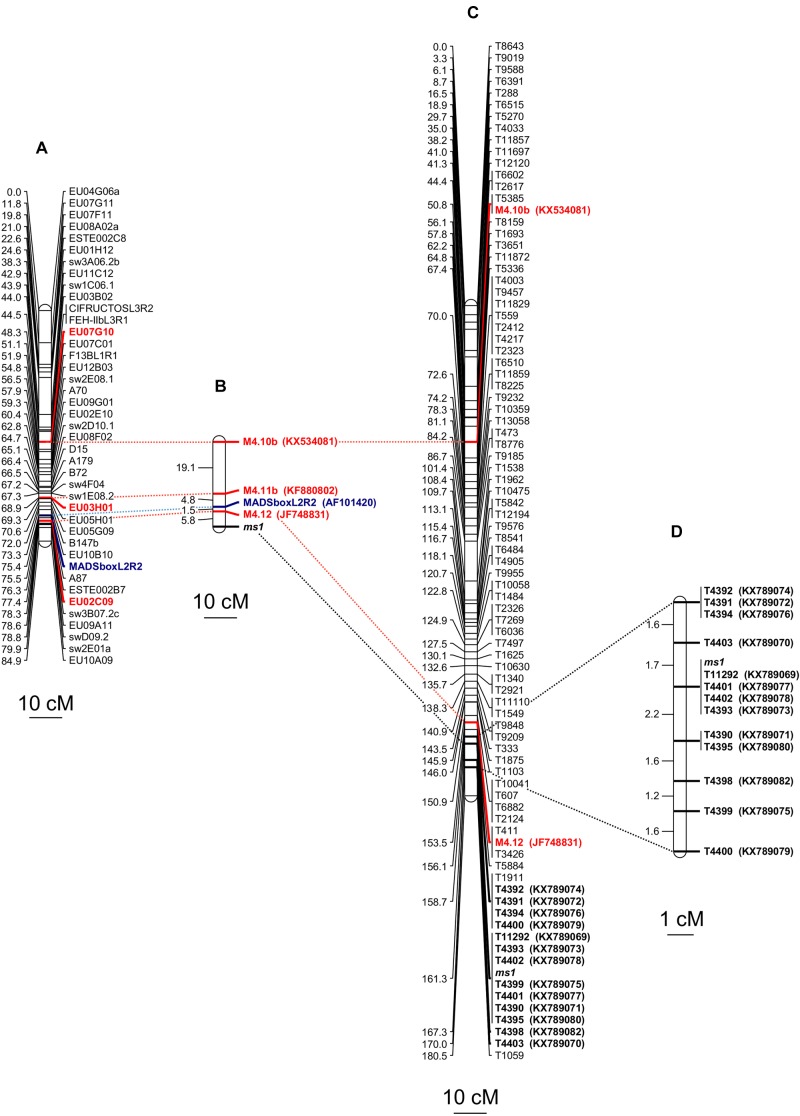
Molecular markers mapped on linkage group 4 and found associated to the male sterility locus (*ms1*) of *C. intybus*. **(A)** Linkage group 4 constructed from Cadalen et al. (2010). **(B)** Genetic distances, expressed in cM, among three SSR markers (in red), one CAPS marker derived from the MADS-box region (in blue) and the male-sterility locus, whose estimates were calculated using a BC_1_ population (198 samples) segregating 1:1 for the male-sterility trait. The SSR markers were retrieved from Ghedina et al. (2015) while the CAPS marker was developed in the present work. **(C)** Linkage group 9 [corresponding to linkage group 4 of Cadalen et al. (2010)] constructed using the data from the Genotyping-By-Sequencing (GBS) approach and based on 44 samples of the BC_1_ population mentioned above. In red are reported the two SSR already used for the first SSR-approach, in bold are listed the 13 SNP showing ≤3 recombinants with the *ms1* locus. **(D)** Chromosomal region around the *ms1*, considering the recombinant data of the aforesaid 13 SNP and a total number of 108 BC_1_ samples.

The fine genetic mapping of three selected SSR markers (Table 1) was successfully pursued and the genetic recombination estimates were validated using 198 BC_1_ individual plants of the segregating population on the basis of chi-square values against independent assortment patterns (Supplementary Table S1). The genetic distances between the male-sterility gene and the microsatellite markers M4.12 (EU02M09) and M4.11b (EU03H01) mapped apart from the *ms1* locus were equal to 5.8 and 12.1 cM, respectively. An additional mapped microsatellite marker, M4.10b (EU07G10), belonging to the same linkage group, was located downstream the *ms1* locus at a genetic distance of 31.2 cM (Figure 2A,B). The DNA sequences of the genomic regions containing these SSR markers were deposited in GenBank as accessions KX534081, KF880802, and JF748831. According to the nomenclature of Cadalen et al. (2010), the gene responsible for male-sterility was associated to linkage group 4, as predicted by Barcaccia and Tiozzo Caenazzo (2012, 2014).

Sequence data published by Galla et al. (2016) on the genome draft of leaf chicory were used to discover, predict and annotate all genes of the genomic contigs encompassing the three molecular markers used for the SSR analysis. Regarding M4.12 (Ghedina et al., 2015), the mapped marker closest to the *ms1* locus, its sequence was found to match with contig_84164, long 36,250 nucleotides and putatively linked with gene models AT3G11330, AT4G03600, AT5G50170, and AT5G63130 (Table 2). It is worth noting that the former one encodes for PIRL9, a member of the Plant Intracellular Ras-group-related LRRs (Leucine rich repeat proteins) and is required for differentiation of microspores into pollen grains.

**Table 2:** List of markers that, aligning against contigs of the first genome draft (Galla et al., 2016), were functionally annotated on the bases of matches with *Arabidopsis* protein database (TAIR10).

Marker map reference	GenBank ID	Marker GenBank ID	Marker localization	Gene model name	Predicted function
E02M09^3^ M4.12^2^	KX639717	JF748831^3^	Upstream	AT5G50170	C2 calcium/lipid-binding and GRAM domain containing protein
	KX639715		Upstream	AT3G11330	Plant Intracellular Ras group-related LLR (PIRL)
	KX639716		Upstream	AT5G63130	Octicosapeptide/Phox/Bem1p family like protein
	KX639719		Upstream	AT5G62230	ERECTA like protein
MADs box L2/R2^1^	KX639714	AF101420^1^	Included	AT4G24540	Agamous-like 24 (AGL24)
CAPS marker^4^	KX639718	KX5455840/1^4^	Upstream (5′ UTR)	AT2G22540	SVP like protein
EU03H01^1,3^ M4.11b^2^	KX639711	KF880802^1,2,3^	Downstream	AT1G01620	Plasma membrane intrinsic like protein
EU07G10^1^ M4.10b^2^	KX639712	KX534081^4^	Upstream	AT5G39000	Malectin/receptor-like protein kinase family protein
T11292^4^	na	KX789069^4^	Downstream	AT3G61700	Helicase with zinc finger domain
T4403^4^	na	KX789070^4^	Downstream	AT4G27280	Calcium-binding EF-hand family protein
T4390^4^	na	KX789071^4^	na	nd	na
T4391^4^	na	KX789072^4^	Included (CDS-synonymous)	AT5G47910	NADPH/respiratory burst oxidase protein D (RBOHD)
T4393^4^	na	KX789073^4^	na	nd	na
T4392^4^	na	KX789074^4^	Downstream	AT5G55350	MBOAT (membrane bound *O*-acyltransferase) family protein
T4399^4^	na	KX789075^4^	Upstream	AT5G61890	ERF subfamily B-4, from ERF/AP2 transcription factor family.
T4394^4^	na	KX789076^4^	na	nd	na
T4401^4^	na	KX789077^4^	Downstream	AT3G61420	BSD domain (BTF2-like transcription factors, Synapse-associated proteins and DOS2-like proteins)
T4402^4^	na	KX789078^4^	Included (CDS-non-synonymous)	XP_022024718^∗^	Transposase of the MuDR family
T4400^4^	na	KX789079^4^	Included (intron)	AT1G15740	Leucine-rich repeat family protein
T4395^4^	na	KX789080^4^	Upstream	AT5G56120	RNA polymerase II elongation factor
T4398^4^	na	KX789082^4^	Included (CDS- synonymous)	AT3G25620	ABC-2 type transporter family protein (ABCG9)

*^1^Cadalen et al., 2010; ^2^Ghedina et al., 2015; ^3^Barcaccia and Tiozzo Caenazzo, 2012, 2014; ^4^Present paper; ^∗^For T4402 is reported the best match with the NR database; na, not available; nd, no significant match detected. Localization of the marker within the genic region is also reported.*

Concerning the MADS-box L2/R21 gene (AF101420) as candidate, the alignment of sequences of part of its 5′-UTR region, exon 1 and the early region of intron 1 recovered from both male-sterile mutants and wild-type plants enabled to map the MADS-box locus on the linkage group 4 (Cadalen et al., 2010) by means a CAPS markers. In fact, three SNPs determining a restriction site were discovered in the amplified sequence of the first exon of the MADS-box gene. The cleavage site of the six-base cutter *Nco*I endonuclease was found to include a polymorphism at position 61 of the nucleotide sequence of the male fertile genotype when compared with the male sterile genotype (GenBank accessions KX455840 and KX455841). The amplification-restriction protocol for the detection of the CAPS marker alleles was applied to the total 198 BC_1_ individual plants of the mapping population. In particular, 14 individuals scored recombinant genotypes when compared with the male-sterile and male-fertile phenotypes, so that the MADS-box gene was mapped at a genetic distance of 7.3 cM apart from the *ms1* locus (Figure 2A,B). A genomic sequence, corresponding to contig_95308, long 8,023 nucleotides, was found to match with the sequence encompassing the CAPS marker that, from a BLASTX approach with the TAIR database, proved to be annotated as AT4G24540, encoding for a protein involved in flowering (Table 2).

### The First SNP-Based Linkage Map of Chicory Through GBS

**FIGURE 3:**
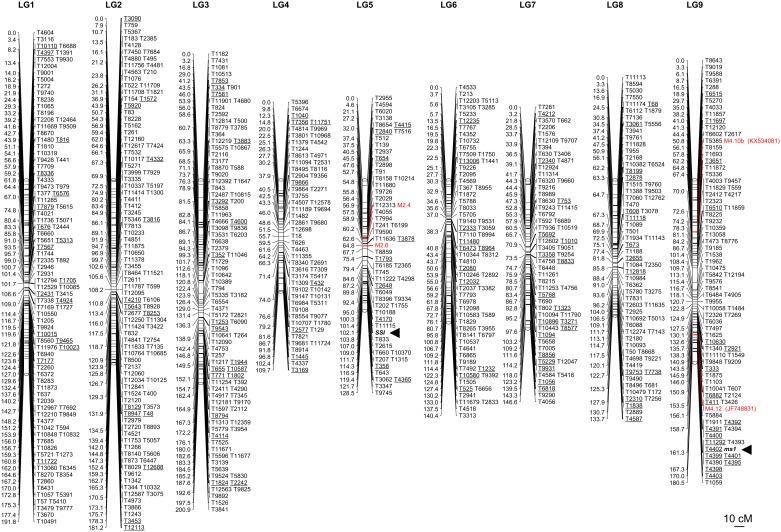
The first SNP-based linkage map in *C. intybus*. Tags underlined represent those mapped sequences showing a significant match (BLASTN, similarity >90%, *E*-value < 1E-50) with contigs from the first genome draft of chicory (Galla et al., 2016) that in turn aligned against protein from the TAIR database (BLASTX, *E*-value < 1E-5). The correspondence between each underlined tag and the best TAIR match is reported in Supplementary Table S3. The male-sterility locus (*ms1*) was assessed by recording the target locus as a putative gene fully co-segregating with the trait. Four SSR markers, M4.10b, M4.12 [from linkage group 4 of Cadalen et al. (2010)], M2.6 and M2.4 [from linkage group 2 of Cadalen et al. (2010)] were used to genotype the same samples employed for the SNP-based map and integrated in the linkage map. M4.10b and M4.12 were chosen because co-segregating with the *ms1* (Barcaccia and Tiozzo Caenazzo, 2012, 2014), M2.6 and M2.4 were selected because were found to be associated with the sporophytic self-incompatibility (SSI) locus by Gonthier et al. (2013).

A GBS approach was applied to a subset of the BC_1_ population, consisting of 22 male sterile and 22 male fertile plants, in order to build the first SNP-based linkage map in *Cichorium* spp. and to identify markers associated with the *ms1* locus. NextSeq 500 v2 Illumina platform produced 419,884,246 raw reads. After quality and adapter trimming, we obtained 339,081,743 reads that were used to create a reference catalog of 1,192,451 consensus loci. A raw pool of 16,353 SNPs was identified using Freebayes v1.0.2-16. After removal of (1) SNPs with more than 30% of missing data, (2) SNPs with a sequence depth ≤ 8×, (3) tri- and tetra-allelic SNPs, and (4) SNPs with allele frequencies across all samples ≤5% and ≥95%, 1,995 SNPs were retained for the construction of a genetic linkage map. A total of 727 SNPs clustered and mapped into 9 linkage groups spanning a total length of 1,413 cM (Figure 3 and Supplementary Table S2). Each mapped SNP-carrying read was used to anchor the first genome draft of leaf chicory by conducting a default BLASTN analysis. A total of 688 out of 727 genetically mapped reads (95%) strongly matched (similarity >90%, *E*-value < 1E-50) sequences in at least one genomic contig. Considering top hits only, 3.7 Mb (0.3% of the whole genome) of the chicory genome sequence was anchored. Among the mapped contigs, 18.6% aligned with expressed regions from the TAIR Database (BLASTX, *E*-value < 1E-5). This allowed organizing 128 coding regions over the 9 linkage groups (Supplementary Table S3). Among them, it was possible to identify 46 different enzymatic proteins, 8 membrane proteins and 4 transcription factors.

The *ms1* locus, along with the M4.10b and M4.12 SSR markers, mapped to linkage group 9 of our genetic map (Figure 2C, 3), allowing us to associate it with the linkage group 4 from Cadalen et al. (2010) (see also Figure 2A). In particular, marker M4.12 co-segregated with the target gene and it was mapped at 7.8 cM from the *ms1* locus (Figure 2C). Thirteen SNPs exhibited ≤3 recombination events with the target *ms1* locus, seven of which (T11292, T4393, T4402, T4399, T4401, T4390, and T4395) cosegregated with *ms1* in the population of 44 BC_1_ progeny (Figure 2C). The GBS reads corresponding to these 13 markers have been deposited in GenBank under accession numbers KX789069-KX789080, KX789082. Ten of the chicory contigs carrying the mapped SNPs showed a significant match (*E*-value < 10-5) with TAIR database (Table 2 and Supplementary Table S3).

Since a recent study located the SSI locus (Gonthier et al., 2013) in linkage group 2 from Cadalen et al. (2010), two SSR markers from this group (namely M2.6 and M2.4) were used to genotype the BC_1_ samples employed for the GBS strategy. This analysis allowed us to associate the S-locus of leaf chicory to our linkage group 5 and the genetic distance between the two SSR markers resulted equal to 7.9 cM (Figure 3).

### Validation of the SNPs Linked to the *ms1* Locus

The map positions of the 13 male sterility-associated SNPs were validated by analyzing an additional 64 BC_1_ progeny (32 male sterile and 32 male fertile). This brought the number of BC_1_ progeny analyzed to 108, including the initial pool of 44 BC_1_ samples analyzed by GBS. For each of the SNP markers, two pairs of primers targeting the two alleles were used in separate reactions. Because BC_1_ progeny are either heterozygous or homozygous for the recurrent parent allele, the primer set that amplified the recurrent parent allele generated amplification products in all BC_1_ progeny and hence acted as positive control. The other primer set amplified the alternate allele which was present only in heterozygous progeny (Supplementary Figure S1). Linkage analysis across the 108 progeny showed that all 13 SNPs mapped to a 9.9 cM region on linkage group 9 [corresponding to linkage group 4 according to Cadalen et al. (2010), Figure 2D]. Four SNPs (T11292, T4393, T4401, and T4402) co-segregated with the *ms1* locus (Figure 2D and Supplementary Table S4).

The GBS reads carrying the SNP markers T11292 (KX789069) and T4401 (KX789077) aligned against contig_55191 and contig_71514 in the chicory draft genome assembly (Galla et al., 2016). These contigs had significant matches with gene models AT3G61700 (contig_55191) and AT3G61420 (contig_71514) (Table 2 and Supplementary Table S3) that encoded, respectively, for a helicase with zinc-finger protein and a BSD domain (BTF2-like transcription factors, synapse-associated proteins and DOS2-like proteins). The SNPs were located downstream from the coding region. The read carrying SNP T4402 (KX789078) aligned with contig_2496, but this sequence did not show any significant BLAST hits with proteins in the TAIR database. Extending the BLASTX analyses to NCBI’s NR database, contig_2496 was found to match significantly (*E*-value = 1E-43, Similarity 44%) with a locus from *Helianthus annuus* (Asteraceae family) encoding for a transposase of the MuDR family (XP_022024718). Moreover, the discriminant SNP resulted to be non-synonymous and it was located in the zinc-finger domain (ZNF_PMZ) of the protein. The read carrying SNP T4393 (KX789073) did not show any significant match with either TAIR or the NR database.

### Micro-Synteny Relationships Between *L. sativa* and *C. intybus* Narrow Chromosomal Segments Enabled the Isolation of MYB103 as Candidate Gene for Male-Sterility

**FIGURE 4:**
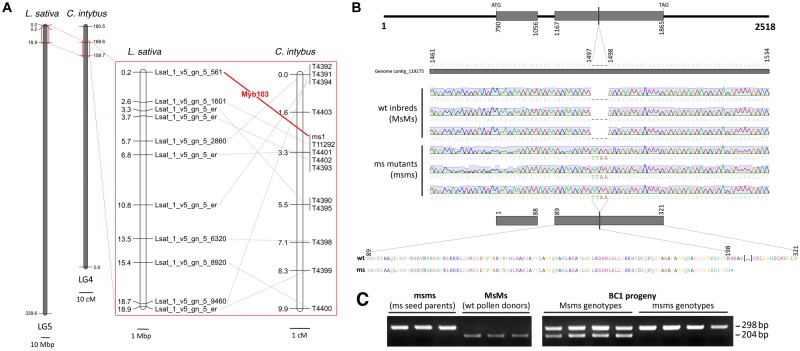
Analysis of MYB103 in *C. intybus*. **(A)** Micro-mesosynteny between a peripheral region of chromosome 5 of *L. sativa* and a DNA region of 9.9 cM around *ms1* locus of *C. intybus*, suggested a possible involvement of MYB103 in the male sterility condition. **(B)** The alignment of eight MYB103 sequences – four sequenced from male fertile (wt) unrelated accessions and four from male sterile accessions (ms), highlighted an insertion of four bases in the second exon of the latter group. The insertion introduced a pre-mature stop codon and the predicted protein resulted 123 amino acids shorter (i.e., 198 amino acids long rather than 321 long for the wt protein). **(C)** Example of the AS-PCR profiles generated in the male sterile mutants (ms), male fertile wild-type (wt) parents and a representative subset of 8 BC1 progeny plants (segregating 1 Msms : 1 msms) with the allele-specific primers for MYB103.

A BLASTN approach, performed using the lettuce genome as database, highlighted that 10 out of the 13 contigs carrying the *ms1*-associated SNPs, aligned (*E*-value < 1E-20) with as many loci of *L. sativa*, all located in a peripheral region of ∼18 Mbp of chromosome 5 (Figure 4A). In the same chromosomal region of *L. sativa* mapped a transcription factor from the MYB family, known as MYB103 and functionally required for anther development in *Arabidopsis thaliana* (Zhang et al., 2007). From a new BLASTN alignment, carried out using the lettuce genome as database, contig_119275 showed the 92% of similarity (*E*-value = 0.0) with the MYB103 locus of *L. sativa* (namely Lsat_1_v5_gn_5_561). Since a reciprocal BLASTN search accomplished using the chicory genome draft as database and Lsat_1_v5_gn_5_561 as query confirmed the match (91% similarity, *E*-value = 0.0), the CDS region entirely included within contig_119275 was considered the putative orthologous of MYB103 in chicory. The DNA segment carrying the two exons of the gene as well as the intron between them was amplified by target-sequence PCR and Sanger-sequenced in eight chicory accessions (4 male sterile - namely D49, A89, B86, D17 – and four male fertile inbreed lines, namely 2111, 202, 231, and 334) using a strategy of overlapping primer walking. The multiple sequence alignment enabled to detect an insertion of four nucleotides (TTAA) in position 1497 of the contig, within the second exon of the male sterile individuals (Figure 4B). Moreover, based on the AS-PCR assay (Figure 4C) performed using 64 BC_1_ accessions (32 male sterile and 32 male fertile), MYB103 was found to fully co-segregate with *ms1* and no recombinants were detected. Sequence data of the entire MYB103 gene were deposited in GenBank with accession no. MK285053–MK285054.

## Discussion

To the best of our knowledge, this is the first time that male-sterile mutants have been genetically characterized in leaf chicory (*C. intybus* subsp. *intybus* var. *foliosum*). The main goals of our study were to build the first SNP-based linkage map in this species and to accomplish the molecular mapping of the male-sterility trait.

A deep comprehension of nuclear male-sterility systems is extremely important for their exploitation in plant breeding, as male-sterility is one of the most effective methods to produce F_1_ hybrid varieties in crop plants (Rajeshwari et al., 1994; Havey, 2004; Barclay, 2010; Acquaah, 2012). F_1_ hybrids are usually developed by crossing two highly homozygous parental lines, selected to obtain highly heterozygous progeny, which are characterized not only by high uniformity of phenotypic traits, but also by strong heterosis in terms of productivity.

A recent cytological study of a naturally occurring male-sterile mutant in leaf chicory has shown that micro-sporogenesis proceeds regularly up to the development of tetrads (Barcaccia and Tiozzo Caenazzo, 2012, 2014). After that, all microspores arrest their developmental program. At the beginning of micro-gametogenesis, non-viable shrunken microspores were clearly visible within anthers. Moreover, detailed investigations indicated the occurrence of meiotic abnormalities in the male-sterile mutants, especially at prophase I. Abnormal pairings and chromosomal loops were observed during pachytene. This new mutant, whose male-sterility is caused by a recessive nuclear gene, has been applied in the production of F_1_ hybrids of Radicchio, and has been recently subjected to patenting (Barcaccia and Tiozzo Caenazzo, 2012, 2014). However, beyond the fact that the NMS was discovered and mapped in root chicory (Desprez et al., 1994; Gonthier et al., 2013) and leaf chicory (Barcaccia and Tiozzo Caenazzo, 2012, 2014) within linkage groups 5 and 4, respectively, no genetic information is available about this locus.

Exploiting an SSR-based approach, the gene responsible for male-sterility in leaf chicory was found genetically linked to the genomic locus M4.12 (JF748831), an AFLP-derived amplicon encompassing an SSR region, about 5.8 cM apart from the *ms1* locus. Two additional SSR markers, corresponding to genomic loci M4.10b (KX534081) and M4.11b (KF880802), were mapped in the same linkage group at a genetic distance equal to, respectively, 31.2 and 12.1 cM from the *ms1* locus. Although mapped on a chromosomal region surrounding the gene of interest, the three discovered SSR markers were found loosely linked to the *ms1* gene. Considering the functional annotation of the three SSR-containing genomic sequences, E02M09, the closest marker to the *ms1* locus, was found to be putatively linked with a TAIR gene (AT3G11330, PIRL 9 protein) required for differentiation of microspores into pollen grains (Forsthoefel et al., 2013), which definitely is the phenomenon described as disrupted in our *ms1* mutants based on cytological observations (Barcaccia and Tiozzo Caenazzo, 2012, 2014). However, the relatively high number of recombinants detected in the BC_1_ population, supporting a genetic distance of 5.8 cM from the *ms1* locus, proved that it could not be responsible for male-sterility in leaf chicory.

The diagnostic CAPS marker derived from the MADS-box L2/R2 gene (KX455840–KX455841) was found genetically linked at 7.3 cM apart from the *ms1* locus. This marker encompasses a genomic block that includes a MADS-box protein (AT4G24540) and a protein that acts as a floral repressor (AT4G22540). Functional analyses by molecular genetic studies in model eudicots, such as *A. thaliana* L., have shown that the proteins encoded by these two genes are both essential for the regulation of various aspects of flower development (Yamaguchi and Hirano, 2006) but no information about their involvement in the male-sterility mechanism is available. Moreover, the relatively high number of recombinants detected in the BC_1_ population raises some doubts regarding its role in the pollen development in leaf chicory.

A genotyping-by-sequencing approach allowed us to construct the first SNP-based linkage map and to narrow down the genomic window around the *ms1* locus in leaf chicory. A total of 727 reads-carrying SNPs were clustered into 9 linkage groups. The first genome draft of leaf chicory was then used to anchor 688 contigs and 128 coding regions to the genetic map. Among the enzymes mapped, it was possible to identify proteins involved in the biosynthesis of *N*-glycan (AT5G19690), cutin, suberin and wax (AT5G55340), diterpenoid (AT5G25900) and amino acids, including valine, leucine and isoleucine (AT1G31180). Other enzymes resulted specifically involved in metabolism processes like glycine, serine and threonine metabolism (AT4G29840), glyoxylate and dicarboxylate metabolism (AT2G05710), inositol phosphate (AT5G42810) and ascorbate and aldarate metabolism (AT5G56490). Two different proteins VAMP713 (AT5G11150) and VAMP714 (AT5G22360), with a key role in the vacuolar trafficking during salt stress (Leshem et al., 2006) were mapped in the linkage groups 7 and 8, respectively. Finally, a noteworthy SNP marker mapped in the linkage group 9 was associated to a genomic contig that, in turn, matched with an ERF BUD ENHANCER in *Arabidopsis* (EBE, AT5G61890). This transcription factor, member of the APETALA2/ETHYLENE RESPONSE FACTOR (AP2/ERF) transcription factor superfamily, was found to promote cell proliferation, leading to enhanced callus growth, to stimulate axillary bud formation and outgrowth, and to affect shoot branching, acting in cell cycle regulation and dormancy breaking (Mehrnia et al., 2013).

Curiously, 22 mapped contigs matched with as many mitochondrial genes of *Arabidopsis* and, in particular, ATMG00300 (14 matches with as many contigs) and ATMG00 750 (3 matches with as many contigs). From TAIR database they resulted annotated as ‘Gag-Pol-related retrotransposon family protein’ and ‘Gag-Pol-Env polyprotein,’ respectively. We found that these two classes of mitochondrial retrotransposons were detected in multiple copies throughout the nuclear genome of several species. At this regards, according to what reported in GenBank database, at least three ATMG00300-like copies were located within chromosomes 3, 7, and 15 of *Malus domestica* as well as in the linkage groups 2, 5, and 6 of *Glycine max.* In *A. thaliana*, the same locus was found also within chromosome 2. This is in accordance with large and unexpected organellar-to-nuclear gene-transfer events highlighted in species like rice (Ueda et al., 2005) and Arabidopsis (Lin et al., 1999). Conversely, we cannot exclude an opposite situation where transposable elements were originally transferred from the nuclear genome to the mitochondrial one as highlighted in *Malus x domestica* (Goremykin et al., 2012). However, the abundance of retroelements sequences identified within the chicory map (e.g., 24 out of 128 coding regions were retrotransposon family proteins) is coherent with what already reported in other species like rice or maize where the retroelements represented, respectively, 14% (Sasaki et al., 2002) and 49% (Meyers et al., 2001) of the whole genome.

Two SSR markers (M2.6 and M2.4), mapped on the linkage group 2 by Cadalen et al. (2010), known for carrying the SSI locus (Gonthier et al., 2013) in root chicory, were used to genotype the 44 samples employed for the GBS. This analysis enabled to associate the self-incompatibility locus to the linkage group 5 of leaf chicory. The genetic distance between these two mapped SSR markers was 7.9 cM whereas they were 14.9 cM apart in the genetic map developed by Cadalen et al. (2010).

Recording the target *ms1* locus as a putative gene fully co-segregating with the trait mapped and using two SSR markers [i.e., M4.10b and M4.12, according to Ghedina et al. (2015)] co-segregating with the *ms1*, enabled to overlap our linkage group 9 with linkage group 4 from Cadalen et al. (2010) (see Figure 2A,B). At least 13 SNPs were found tightly linked to the target locus, exhibiting three or less recombinants (Figure 2C). In particular, seven out of 13 SNPs (i.e., T11292, T4393, T4402, T4399, T4401, T4390, and T4395) did not show recombinants. To increase the robustness of this finding, an AS-PCR assay was developed focusing on these 13 loci and increasing the number of BC_1_ samples essayed up to 108. This new round of analysis proved to be fast, cheap and highly efficient and, most importantly, allowed us to finely map all the SNPs in a chromosomal DNA region spanning 9.9 cM (Figure 2D) in length, both upstream and downstream the *ms1* locus. The allelic variants of four SNPs proved still to fully co-segregate with the target trait and the corresponding reads were mapped at 0 cM from the *ms1*. Interestingly, two of these genomic sequences, namely T11292 (KX789069) and T4401 (KX789077), retrieved a significant match with two TAIR gene models: a helicase with zinc-finger protein (AT3G61700) and a BSD domain (BTF2-like transcription factors, synapse-associated proteins and DOS2-like proteins, AT3G61420) characterizing the RNA polymerase II transcription factor B. The fact that these two genes resulted to be strictly associated also in the chromosome 3 of *A. thaliana* (∼100 kb one from the other), strengthens the possibility that they may interact synergistically. Moreover, according to Honys and Twell (2004), both genes resulted differentially expressed during micro-gametogenesis. Nevertheless, no information about their involvement in the male-sterility mechanism is available and the position of the two SNPs downstream of the coding regions raises some doubts regarding their role in the pollen development.

Patterns of conserved synteny from the genomes of different organisms play a central undertaking in the field of molecular biology (Veltri et al., 2016). At this regard, the 13 contigs carrying the SNPs associated to the *ms1* locus were aligned against the lettuce genome, currently considered the reference assembly for the Asteraceae family (Reyes-Chin-Wo et al., 2017). Ten of these contigs significantly matched with as many loci mapping in a small peripheral region, representing the 5.5% of chromosome 5 of *L. sativa*. It is worth noting that the 10 contigs of chicory spanned a chromosome region of 9.9 cM, constituting again the 5.5% of linkage group 4 of *C. intybus*. Looking at the micro mesosynteny observed between these two species – defined as the conservation of gene content but not gene order or orientation (Ohm et al., 2012) – transcription factor MYB103 stood out as the most interesting locus to investigate. In fact, according to Zhang et al. (2007), a mutation in the first exon of MYB103 in *A. thaliana* is responsible for a male sterile phenotype. In particular, the tapetum development and callose dissolution resulted altered in MYB103 defective plants. Moreover, most of the microspores in mature anthers were degraded and surviving microspores lacked exine. This is totally in agreement with the cytological findings reported in chicory by Barcaccia and Tiozzo Caenazzo (2012, 2014) as well as in other crops like wheat, rice, canola, and cotton (Phan et al., 2012). A BLASTN approach enabled to identify the orthologous of MYB103 in chicory and the nucleotide similarity with the same gene in lettuce resulted very high (91%, *E*-value = 0.0). From a BLASTX alignment, the amino acid similarity resulted very high (90%, *E*-value = 1E-84) also between the putative MYB103 of chicory and the orthologous gene in *A. thaliana*. The sequence conservation observed among these three species is coherent with the conservation of the gene function already reported by Phan et al. (2012). Preliminary analyses carried out on eight unrelated accessions (four male sterile and four male fertile inbreed samples) highlighted within the male sterile group, the occurrence of a four nucleotides insertion in the second exon of the putative CDS (see Figure 4). The insertion introduced a pre-mature stop codon in the MYB103 coding sequence and the protein predicted *in silico* resulted 123 amino acids shorter (i.e., 198 amino acids long for the mutants rather than 321 as for the wild types). Zhang et al. (2007) highlighted that mutations of MYB103 negatively affect the expression of both A6 – a putative β-1,3-glucanase involved in callose dissolution – and MS2 – a fatty acid reductase putatively involved in sporopollenin synthesis and therefore in exine formation. Our hypothesis is that a truncated variant of MYB103, lacking more than a third of the amino acid sequence, may affect the process of pollen development in leaf chicory. As a matter of fact, we demonstrated that MYB103 fully co-segregates with *ms1*, further corroborating the evidence that it could be the responsible for the male sterility in *C. intybus*.

## Conclusion

The male-sterility gene (*ms1*) of leaf chicory was firstly confined to a chromosomal region spanning 5.8 cM through an SSR-based approach. The construction of a SNP-based linkage map and the application of an AS-PCR assay enabled to narrow down the genomic window around the target locus and to select SNPs mapped at 0 cM from *ms1* locus. Moreover, the newly developed genetic linkage map combined with the micro-mesosynteny analysis enabled to identify MYB103, a gene encoding for a transcription factor of the MYB family, fully co-segregating with the *ms1* locus, which could be considered a primary candidate gene for male-sterility.

## Data Availability

The datasets generated for this study can be found in GenBank (Accession Numbers: KX789069, KX789070, KX789071, KX789072, KX789073, KX789074, KX789075, KX789076, KX789077, KX789078, KX789079, KX789080, KX789082, MK285053, and MK285054).

## Author Contributions

FP and GB designed the research. FP and VP conducted and controlled the experiments. PQ carried out the bioinformatics analyses. FP and KD analyzed data. FP, GB, and KD wrote the manuscript. All authors contributed to editing the manuscript.

## Conflict of Interest Statement

The authors declare that the research was conducted in the absence of any commercial or financial relationships that could be construed as a potential conflict of interest.

## Footnotes

1 http://bio-bwa.sourceforge.net/

2 https://github.com/ekg/freebayes#readme

3 http://rna-informatics.uga.edu/malmberg/rlmlab
