# Supplementary material for: Construction of the First SNP-Based Linkage Map Using Genotyping-by-Sequencing and Mapping of the Male-Sterility Gene in Leaf Chicory
Source: Front Plant Sci. 2019 Mar 11;10:276. doi: 10.3389/fpls.2019.00276 (PMC6421318; doi:10.3389/fpls.2019.00276)
Supplement: Supplementary file 3 [file Table_3.DOCX]

**Table 3S.** List of GBS-derived tags mapped over the 9 linkage groups (LG) of *Cichorium intybus* subsp. *intybus* var. *foliosum* and hypothetic function on the basis of matches with *Arabidopsis* protein database (TAIR database). Tags underlined were found to be associated at 0cM with the male sterility locus (*ms1*) based on 108 samples. For T4402 (LG9) is reported the best match with the NR database.

| \| **LG1** \| \| **LG2** \| \| **LG3** \| \| **LG4** \| \| **LG5** \| \| **LG6** \| \| **LG7** \| \| **LG8** \| \| **LG9** \| \| \| --- \| --- \| --- \| --- \| --- \| --- \| --- \| --- \| --- \| --- \| --- \| --- \| --- \| --- \| --- \| --- \| --- \| --- \| \| **Tag** \| **TAIR** \| **Tag** \| **TAIR** \| **Tag** \| **TAIR** \| **Tag** \| **TAIR** \| **Tag** \| **TAIR** \| **Tag** \| **TAIR** \| **Tag** \| **TAIR** \| **Tag** \| **TAIR** \| **Tag** \| **TAIR** \| \| T10110 \| AT4G23160 \| T3090 \| AT5G27870 \| T7853 \| AT4G22270 \| T1040 \| AT1G63800 \| T4415 \| ATMG00300 \| T12235 \| AT5G40170 \| T4212 \| AT3G04650 \| T68 \| AT2G27920 \| T6515 \| AT4G32090 \| \| T4397 \| AT1G59830 \| T1572 \| AT3G47680 \| T334 \| ATMG00820 \| T7356 \| AT1G20960 \| T2840 \| AT4G23160 \| T13006 \| ATMG00300 \| T2340 \| AT2G28550 \| T3061 \| AT5G22360 \| T11697 \| AT4G23160 \| \| T816 \| ATMG00300 \| T5920 \| ATCG00780 \| T7581 \| AT2G17220 \| T11751 \| AT1G12480 \| T654 \| AT4G23160 \| T2333 \| ATMG00750 \| T753 \| AT4G23160 \| T8199 \| AT3G44160 \| T3651 \| AT1G21280 \| \| T8336 \| AT1G41920 \| T4332 \| AT1G31180 \| T3883 \| AT3G25800 \| T9866 \| AT5G37020 \| T3878 \| AT4G23160 \| T11480 \| ATMG00750 \| T5692 \| AT5G20870 \| T2878 \| AT4G23160 \| T6510 \| AT5G48050 \| \| T6576 \| AT5G63540 \| T3816 \| AT4G17250 \| T3292 \| AT3G03960 \| T432 \| AT4G23160 \| T1793 \| AT3G49142 \| T6473 \| ATMG00810 \| T1010 \| AT5G48050 \| T608 \| AT5G48050 \| T10630 \| AT5G48100 \| \| T7879 \| AT4G23160 \| T4210 \| AT4G23160 \| T4600 \| AT4G35160 \| T2577 \| AT2G46495 \| T2648 \| AT5G18200 \| T8964 \| ATMG00300 \| T3358 \| AT4G03230 \| T11118 \| AT5G56490 \| T2921 \| AT1G58200 \| \| T676 \| AT3G21250 \| T5643 \| ATCG00740 \| T352 \| ATMG00300 \| T1445 \| AT5G35160 \| T4170 \| AT1G80350 \| T2080 \| ATMG00300 \| T8833 \| AT5G57140 \| T673 \| AT4G23160 \| T333 \| AT1G79000 \| \| T5313 \| AT1G16800 \| T8253 \| ATMG00300 \| T1253 \| ATMG00810 \| T3169 \| ATMG00750 \| T358 \| AT1G74680 \| T12032 \| ATMG00300 \| T5768 \| AT3G02645 \| T2655 \| AT1G34070 \| T6882 \| AT5G42810 \| \| T7567 \| AT4G29840 \| T8129 \| AT2G19880 \| T9543 \| ATMG00300 \|  \|  \| T4365 \| AT3G29638 \| T1232 \| AT4G23160 \| T1323 \| AT1G31540 \| T12818 \| AT2G05710 \| T411 \| AT5G25900 \| \| T1705 \| AT3G29785 \| T8947 \| AT5G19690 \| T1944 \| AT3G49142 \|  \|  \|  \|  \| T10580 \| AT4G33070 \| T10896 \| AT5G66910 \| T9753 \| AT4G15040 \| T4392 \| AT5G55340 \| \| T2431 \| AT4G23160 \| T48 \| AT4G23160 \| T655 \| ATMG01250 \|  \|  \|  \|  \| T525 \| AT2G41770 \| T3271 \| AT1G17210 \| T7738 \| AT2G33680 \| T4391 \| AT5G47910 \| \| T4924 \| AT4G23160 \| T12688 \| AT5G41980 \| T10587 \| AT4G23160 \|  \|  \|  \|  \|  \|  \| T8577 \| AT3G14590 \| T2310 \| AT5G52520 \| T4400 \| AT1G15740 \| \| T10015 \| ATMG00300 \| T3453 \| AT4G03080 \| T771 \| AT5G48050 \|  \|  \|  \|  \|  \|  \| T1094 \| AT5G11150 \| T1838 \| AT5G05200 \| T11292 \| AT3G61700 \| \| T9465 \| AT1G21580 \| T12113 \| ATMG00300 \| T1802 \| AT3G47210 \|  \|  \|  \|  \|  \|  \| T8856 \| ATMG00300 \| T4587 \| AT2G40280 \| T4402 \| XP_022024718 \| \| T10023 \| AT5G41980 \|  \|  \| T8794 \| AT4G30790 \|  \|  \|  \|  \|  \|  \| T5229 \| AT2G15220 \|  \|  \| T4399 \| AT5G61890 \| \| T7177 \| AT4G23160 \|  \|  \| T4114 \| AT5G20320 \|  \|  \|  \|  \|  \|  \| T9931 \| ATMG00300 \|  \|  \| T4401 \| AT3G61420 \| \| T11722 \| ATMG00300 \|  \|  \| T2242 \| AT3G04350 \|  \|  \|  \|  \|  \|  \| T1056 \| AT5G49630 \|  \|  \| T4395 \| AT5G56120 \| \|  \|  \|  \|  \| T1824 \| ATMG00820 \|  \|  \|  \|  \|  \|  \| T6818 \| AT4G23160 \|  \|  \| T4398 \| AT3G25620 \| \|  \|  \|  \|  \|  \|  \|  \|  \|  \|  \|  \|  \|  \|  \|  \|  \| T4403 \| AT4G27280 \| | |  | |  | |  | |  | |  | |  | |  | |  | |  | |  | |  | |  | |  | |  | |  | |  | |  | |
| --- | --- | --- | --- | --- | --- | --- | --- | --- | --- | --- | --- | --- | --- | --- | --- | --- | --- | --- | --- | --- | --- | --- | --- | --- | --- | --- | --- | --- | --- | --- | --- | --- | --- | --- | --- | --- | --- | --- | --- | --- | --- | --- | --- | --- | --- | --- | --- | --- | --- | --- | --- | --- | --- | --- | --- | --- | --- | --- | --- | --- | --- | --- | --- | --- | --- | --- | --- | --- | --- | --- | --- | --- | --- | --- | --- | --- | --- | --- | --- | --- | --- | --- | --- | --- | --- | --- | --- | --- | --- | --- | --- | --- | --- | --- | --- | --- | --- | --- | --- | --- | --- | --- | --- | --- | --- | --- | --- | --- | --- | --- | --- | --- | --- | --- | --- | --- | --- | --- | --- | --- | --- | --- | --- | --- | --- | --- | --- | --- | --- | --- | --- | --- | --- | --- | --- | --- | --- | --- | --- | --- | --- | --- | --- | --- | --- | --- | --- | --- | --- | --- | --- | --- | --- | --- | --- | --- | --- | --- | --- | --- | --- | --- | --- | --- | --- | --- | --- | --- | --- | --- | --- | --- | --- | --- | --- | --- | --- | --- | --- | --- | --- | --- | --- | --- | --- | --- | --- | --- | --- | --- | --- | --- | --- | --- | --- | --- | --- | --- | --- | --- | --- | --- | --- | --- | --- | --- | --- | --- | --- | --- | --- | --- | --- | --- | --- | --- | --- | --- | --- | --- | --- | --- | --- | --- | --- | --- | --- | --- | --- | --- | --- | --- | --- | --- | --- | --- | --- | --- | --- | --- | --- | --- | --- | --- | --- | --- | --- | --- | --- | --- | --- | --- | --- | --- | --- | --- | --- | --- | --- | --- | --- | --- | --- | --- | --- | --- | --- | --- | --- | --- | --- | --- | --- | --- | --- | --- | --- | --- | --- | --- | --- | --- | --- | --- | --- | --- | --- | --- | --- | --- | --- | --- | --- | --- | --- | --- | --- | --- | --- | --- | --- | --- | --- | --- | --- | --- | --- | --- | --- | --- | --- | --- | --- | --- | --- | --- | --- | --- | --- | --- | --- | --- | --- | --- | --- | --- | --- | --- | --- | --- | --- | --- | --- | --- | --- | --- | --- | --- | --- | --- | --- | --- | --- | --- | --- | --- | --- | --- | --- | --- | --- | --- | --- | --- | --- | --- | --- | --- | --- | --- | --- | --- | --- | --- | --- | --- | --- | --- | --- | --- | --- | --- | --- | --- | --- | --- | --- | --- | --- | --- | --- | --- | --- | --- | --- | --- | --- | --- | --- | --- | --- | --- | --- | --- | --- | --- | --- | --- | --- | --- | --- | --- | --- | --- | --- | --- | --- | --- | --- | --- | --- | --- | --- |
|  |  | |  | |  | |  | |  | |  | |  | |  | |  | |  | |  | |  | |  | |  | |  | |  | |  | |  |
