# Supplementary material for: Construction of the First SNP-Based Linkage Map Using Genotyping-by-Sequencing and Mapping of the Male-Sterility Gene in Leaf Chicory
Source: Front Plant Sci. 2019 Mar 11;10:276. doi: 10.3389/fpls.2019.00276 (PMC6421318; doi:10.3389/fpls.2019.00276)

**Figure 1S**. A summary of the AS-PCR profiles generated in the male sterile mutant (*msms*) and male fertile wild-type (*Msms*) parents and a representative subset of 12 BC_1_ progeny plants (segregating 1 *Msms* : 1 *msms*) with the allele-specific primers for the four diagnostic SNP markers co-segregating with male-sterility. For each SNP marker is indicated the point mutation that discriminates male-sterile and male-fertile plants, the specific length of amplicons and the GenBank accession number


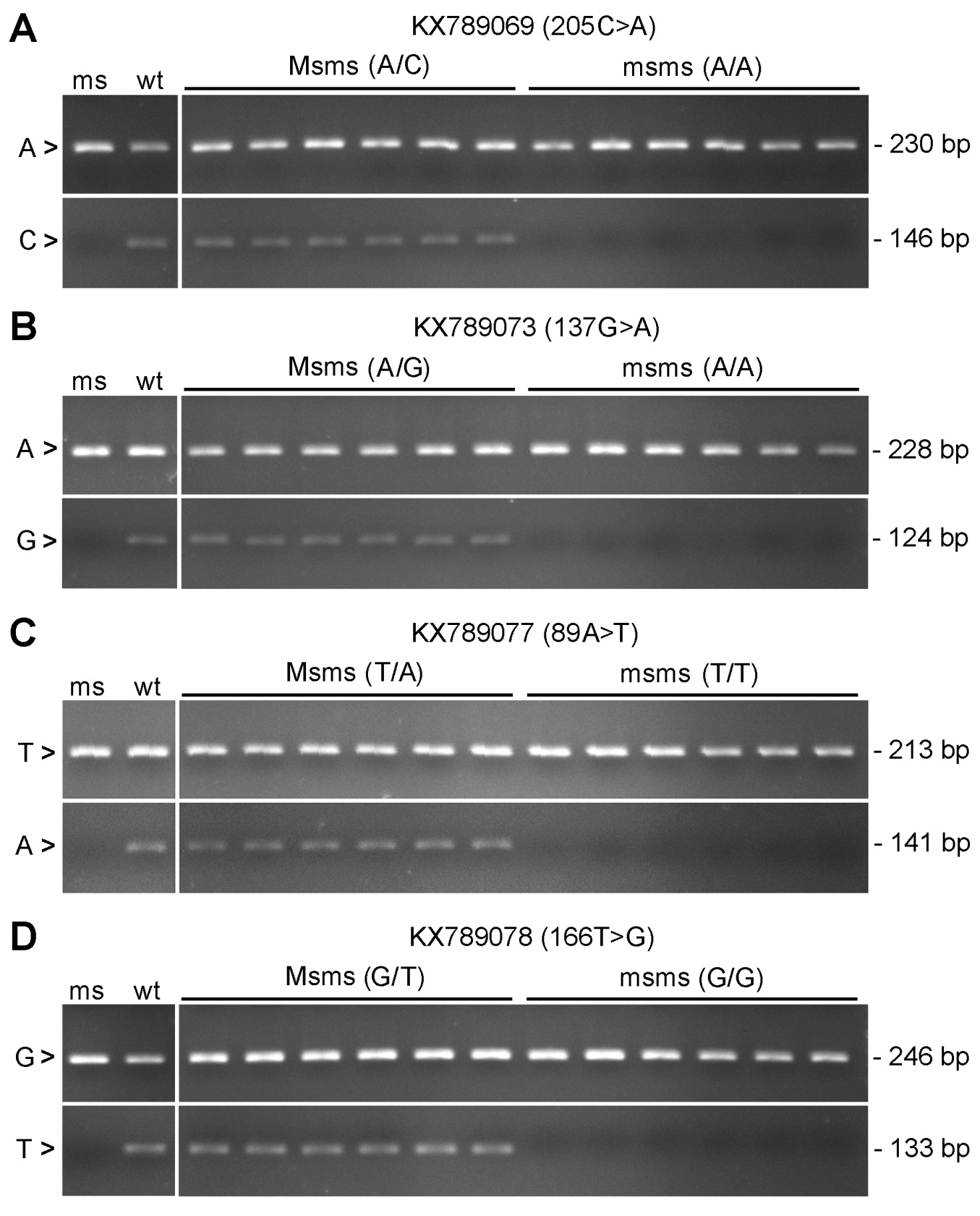

Supplement: Supplementary file 5 [file Data_Sheet_1.docx]
